# Epilepsy and multidimensional time: a phenomenological and neurocognitive perspective inspired by theoretical physics

**DOI:** 10.3389/fpsyg.2026.1884775

**Published:** 2026-08-05

**Authors:** Giancarlo Di Gennaro, Andrea Tomasini, Enrico Michele Salamone

**Affiliations:** 1 IRCCS Neuromed, Pozzilli, Italy; 2 Associazione Italiana Epilessia (Italian Epilepsy Association), Community Council of International Bureau for Epilepsy, Rome, Italy

**Keywords:** epilepsy, intrinsic neural timescales, phenomenology, temporal consciousness, time perception

## Abstract

Recent advances in theoretical physics suggest that time may exhibit a richer, potentially multidimensional structure, challenging classical linear conceptions. This Perspective develops a conceptual framework for understanding altered temporal experience in epilepsy, where seizures—driven by hypersynchronous neuronal activity and disrupted network synchronization—produce both clinical manifestations and striking distortions of subjective time, including déjà vu, jamais vu, temporal acceleration or slowing, looping, and fragmentation. Building on hierarchical models of intrinsic neural timescales, the brain is conceptualized as a dynamic temporal integrator in which distributed cortical regions operate over partially distinct temporal windows to construct coherent experience. Complementary accounts propose that subjective time emerges from the interaction of nested brain–body rhythms spanning multiple frequency bands, providing convergent temporal reference frames for perception and memory. Within this framework, Kletetschka's multidimensional time model is employed strictly as a conceptual analogy to illustrate how partially independent temporal processes may transiently decouple under pathological conditions, without implying a literal neurobiological mapping onto physical time dimensions. Seizure states may therefore be understood as disruptions of temporal integration, revealing alternative modes of temporal organization. Phenomenological analysis further clarifies these dynamics: Husserlian structures of retentio, protentio, and presentatio highlight how epileptic activity can fragment the synthesis of temporal consciousness. Taken together, seizures function as natural experiments in temporal disruption, exposing the plasticity and multi-scale organization of temporal experience. This interdisciplinary synthesis integrates neuroscience, brain–body dynamics, phenomenology, and theoretical physics-inspired analogy to propose that subjective time emerges from coordinated but partially independent temporal processes.

## Highlights

- Seizures may be associated with transitory distortions of subjective time.
- Temporal experience appears to emerge from hierarchical neural timescales, oscillatory coordination, and embodied perception.
- Seizure-related disruptions can transiently reveal alternative, potentially multidimensional temporal organizations.
- Phenomenological frameworks may provide essential insight into altered temporal consciousness.
- Epilepsy can serve as a natural model for investigating the plasticity and plurality of temporal experience.

## 1 Introduction

Time is typically considered linear, flowing from past to future through the present. This assumption underlies everyday experience, language, and conventional neuroscientific models of cognition. However, recent developments in theoretical physics suggest that time may, within specific mathematical frameworks, be described as possessing multiple dimensions or non-linear structures rather than a single unidirectional flow (Kletetschka, 2025). Kletetschka proposes a formal mathematical framework in which time is represented as a three-dimensional manifold, with orthogonal temporal axes that coexist and interact within a unified structure. Importantly, the multidimensional concept of time in physics refers to the fundamental structure of the universe rather than to mechanisms of brain function. In this article, we employ this framework strictly as a conceptual analogy to stimulate reflection on how multiple neural temporal processes might interact in the construction of subjective time. Neural activity unfolds across multiple hierarchical timescales, from fast sensory integration to slower narrative or self-referential processes. Complementary perspectives in neuroscience further emphasize the role of brain–body rhythms in shaping temporal experience (Buzsáki, 2026). According to recent theoretical proposals, experienced time may emerge from the interaction of multiple physiological and neural rhythms that provide reference scales for measuring change. These rhythms span a wide frequency range, from fast neuronal oscillations to slower cycles such as respiration, cardiac activity, and circadian rhythms. Within this framework, subjective time reflects the coordination of these nested temporal processes rather than the output of a single internal clock.

Epileptic seizures provide a unique naturalistic context to investigate these questions. Seizures are transient episodes resulting from abnormally excessive, hypersynchronous neuronal activity (Fisher et al., 2005). At the network level, they involve disruptions in cortical excitability, excitation-inhibition balance, and large-scale synchronization (Fan et al., 2023). Clinically, seizures manifest through a constellation of signs and behaviors that reflect the spatiotemporal evolution of epileptic discharges (Bancaud et al., 1994; Kahane et al., 2006).

Beyond these observable effects, seizures often give rise to profound alterations in subjective temporal experience, including déjà vu, jamais vu, temporal acceleration or slowing, looping, and fragmentation (Kahane et al., 2006; Illman et al., 2012; Hadzic and Andersson, 2024). Importantly, growing evidence suggests that disturbances of temporal processing in epilepsy are not limited to the ictal phase but may also persist during interictal periods in certain epilepsy syndromes. In particular, studies in childhood absence epilepsy have demonstrated impaired temporal judgment outside overt seizure activity, as reflected by abnormalities in time bisection performance and reduced temporal sensitivity. These interictal alterations have been shown to correlate with executive dysfunctions, including deficits in inhibition, processing speed, and cognitive flexibility, pointing to a shared neurophysiological substrate underlying both temporal perception and cognitive control (Cainelli et al., 2019). From this perspective, the multidimensional conceptualization of time provides a framework for interpreting these persistent disruptions as reflecting interactions among partially independent neural temporal processes operating at partially distinct timescales, analogous to Kletetschka's model, rather than as isolated distortions along a single linear timeline. Such findings suggest that disrupted temporal experience in epilepsy may reflect a persistent alteration of neural timing mechanisms rather than a transient epiphenomenon of seizure events.

Notably, fragments of non-linear or multidimensional temporal experience may also transiently emerge in neurologically healthy individuals, as exemplified by phenomena such as déjà vu, indicating that similar neural mechanisms, normally constrained or regulated, may sporadically become manifest even under physiological conditions (Brown, 2003). In this perspective, epileptic activity may be understood as an extreme perturbation that amplifies or unveils intrinsic properties of temporal processing that are otherwise latent.

Such phenomena challenge linear models of temporal consciousness and suggest that subjective time is emergent, context-dependent, and dynamically constructed from neural activity. By Importantly, these oscillatory dynamics are embedded within broader brain–body rhythmic systems. Neural oscillations interact with physiological rhythms such as respiration, cardiac cycles, and circadian modulation, forming nested temporal hierarchies that structure perception and cognition (Buzsáki, 2026). Within this framework, subjective temporal experience may emerge from the coordination of multiple rhythmic reference frames rather than from a centralized neural clock. These experiences could provide a natural window into the mechanisms underlying temporal awareness and may reveal latent variability in human temporal experience that may allow the perception of multiple coexisting temporal representations under certain conditions otherwise inaccessible under normal physiological conditions.

The purpose of this Perspective is not to propose a new mathematical model of temporal processing nor to establish a direct correspondence between physical and neural time. Rather, it aims to integrate converging evidence from clinical epileptology, systems neuroscience, phenomenology, and theoretical physics-inspired concepts into a coherent conceptual framework. By bringing together these complementary perspectives, epilepsy is considered a natural experimental model through which the mechanisms underlying subjective temporal consciousness may become more accessible. The framework is intended to generate empirically testable hypotheses and to stimulate interdisciplinary research rather than to provide a quantitative description of brain dynamics.

## 2 Neurocognitive mechanisms of temporal integration

Neuroscientific research increasingly conceptualizes temporal experience as emerging from distributed neural dynamics rather than a centralized “clock.” Neural activity unfolds across a hierarchy of intrinsic timescales that differ systematically across cortical regions. Primary sensory cortices operate on short timescales, integrating fast-changing inputs, whereas higher-order transmodal networks, such as the default mode network and front oparietal networks, integrate information over longer temporal windows, supporting temporally extended representations (Golesorkhi et al., 2021; Wolff et al., 2022).

These intrinsic timescales act as temporal receptive fields, determining how events are grouped or segregated. Longer integration windows in association cortices allow sequential experiences to be synthesized into coherent narratives, enabling episodic memory, anticipation, and the perception of temporal continuity (Hasson et al., 2008, 2015). Synchronization across thalamocortical loops, hippocampal circuits, and neocortical networks further supports unified temporal perception (Arthuis et al., 2009).

Seizures can destabilize these integrative mechanisms, fragmenting temporal perception and producing overlapping (Arthuis et al., 2009). Changes in excitation-inhibition balance, particularly GABAergic modulation, can alter temporal integration windows, affecting subjective interval estimation (Terhune et al., 2014). Similar distortions in disorders with cortical dysregulation, such as schizophrenia, highlight the general sensitivity of temporal experience to network dynamics (Teixeira et al., 2013). Neuronal oscillations coordinate activity across distributed networks, aligning temporal windows and enabling coherent perception (Buzsáki and Draguhn, 2004; Dehghani et al., 2016). Importantly, these oscillatory dynamics are embedded within broader brain–body rhythmic systems. Neural oscillations interact with physiological rhythms such as respiration, cardiac cycles, and circadian modulation, contributing to nested temporal hierarchies that structure perception and cognition (Buzsáki, 2026). Within this framework, subjective temporal experience may emerge in part from the coordination of multiple rhythmic reference frames rather than from a centralized neural clock. Epileptic seizures, by perturbing these oscillatory and hierarchical mechanisms, may transiently be associated with alternative modes of temporal integration, providing a unique window into the neural construction of temporal experience.

Furthermore, recent studies indicate that network-level plasticity allows functional brain networks to reorganize temporal hierarchies over circadian and multiday scales, contributing to inter-individual variability in seizure phenomenology and linking neural adaptability to subjective time (Laiou et al., 2022; Schroeder et al., 2023).

## 3 Phenomenological perspectives on seizure-induced temporal alterations

In classical phenomenology, Edmund Husserl described temporal consciousness as a dynamic synthesis of retentio (immediate past), protentio (anticipated future), and presentatio (lived present) (Husserl, 1964). According to this framework, subjective time is not composed of discrete moments but emerges from a continuous integration of past retention and future anticipation within present experience. Disruption of this synthesis can produce altered simultaneity, repetition, or temporal dislocation, phenomena frequently reported in epilepsy (Vogeley and Kupke, 2007).

Complementing this framework, Bergson distinguished between quantitative clock time and qualitative lived duration (durée), emphasizing the heterogeneous nature of subjective time (Bergson, 1910). Merleau-Ponty (2005) further proposed that temporality is fundamentally embodied and emerges from the interaction between perception, action, and bodily engagement with the environment. Finally, Minkowski (2022). concept of lived space time continuity (*temps vècu*) highlights continuity and future-directedness as essential features of experience (Minkowski, 2022). Epileptic episodes could transiently disrupt these boundaries, producing altered or non-linear temporal phenomenology. Taken together, these perspectives suggest that seizure-related temporal distortions are not merely pathological errors but structured alterations of the mechanisms that normally integrate past, present, and anticipated future.

## 4 Cultural and historical dimensions of temporal experience

Anthropological research indicates that temporal cognition is shaped not only by neural mechanisms but also by cultural frameworks that organize how past, present, and future are conceptualized (Fulmer et al., 2014; Sircova et al., 2014; Lee et al., 2017). Languages and social practices vary considerably in how they structure temporal relations. Ssorin-Chaikov (2025) further highlights that temporal structuring in different societies often reflects local ecological and social conditions, emphasizing pluralistic and context-dependent conceptions of time.

These cultural variations highlight that subjective time is not solely a biological construct but emerges from the interaction between neural processes and sociocultural context.

In this perspective, seizure-related temporal distortions may be interpreted in relation to pre-existing cultural representations of time. The plurality of temporal perspectives suggests that the temporal anomalies observed in epilepsy may reflect variability in human temporal experience that can be differently interpreted across cultural contexts, amplified by neural disruption rather than solely pathological processes. Recognizing cultural and historical diversity in temporal cognition situates seizure-related phenomena within a broader, context-sensitive understanding of consciousness.

## 5 Discussion

We conceptualize certain subjective ictal symptoms as transient disruptions of temporal integration, providing insight into the neurocognitive and phenomenological mechanisms that structure temporal experience. Seizures can fragment or overlay temporal streams, suggesting alternative modes of temporal organization that are normally stabilized and highlighting the plasticity of temporal consciousness. Insights from theoretical physics, such as multidimensional or non-linear time, offer a conceptual analogy for interpreting these alterations, stimulating hypotheses about how alternative temporal frameworks might emerge from distributed neural dynamics (Kletetschka, 2025). At the neural level, temporal continuity relies on finely tuned network synchronization and hierarchical timescales, with perturbations during seizures exposing both short- and long-timescale contributions. These networks are plastic, reorganizing over circadian and multiday cycles, which may explain inter-individual differences in seizure phenomenology and temporal perception (Laiou et al., 2022; Schroeder et al., 2023).

**Figure 1:**
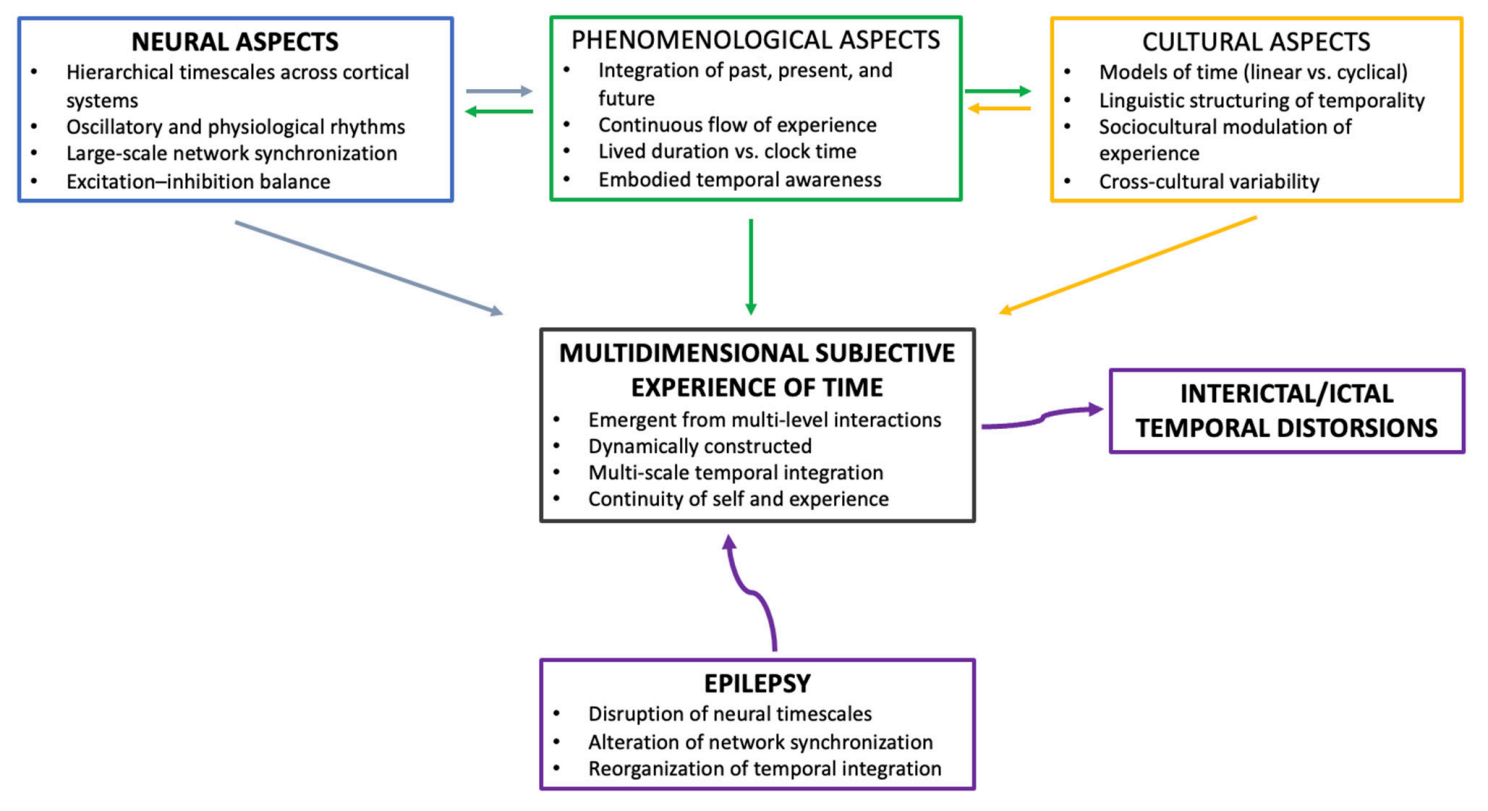
Multidimensional framework of temporal experience. Subjective time emerges from interactions between neural, phenomenological, and cultural dimensions. Epilepsy perturbs neural synchronization and temporal integration, leading to altered subjective temporal experience.

The proposed framework links neural dynamics, subjective experience, and conceptual analogies from physics through three core components: hierarchical neural timescales, brain–body rhythmic scaffolding, and dynamic network integration (Figure 1). Different brain regions integrate information over distinct temporal windows (Hasson et al., 2008; Wolff et al., 2022), while neuronal oscillations interact with physiological rhythms, such as respiration, cardiac cycles, and circadian modulation, providing nested reference frames for structuring experience (Buzsáki, 2026). Coherent temporal perception emerges when these processes synchronize across distributed networks, and seizures transiently disrupt this coordination, producing distorted or overlapping temporal experiences (Arthuis et al., 2009). The present Perspective does not seek to replace existing neuroscientific theories of temporal perception or consciousness. Rather, it proposes an integrative framework in which concepts that have largely evolved in parallel—including intrinsic neural timescales, brain–body rhythmic coordination, phenomenological analyses of lived temporality, and physics-inspired conceptual models—are considered as complementary levels of description. The novelty of the present contribution lies in emphasizing epilepsy as a privileged natural model through which these different perspectives can be examined within a unified conceptual framework.

Phenomenologically, seizures reveal the embodied and context-sensitive nature of temporal experience. Distortions in time perception are not mere errors but structured alterations in how consciousness organizes past, present, and anticipated future (Bergson, 1910; Husserl, 1964; Merleau-Ponty, 2005; Vogeley and Kupke, 2007; Minkowski, 2022). This multidimensional temporal framework aligns with dominant theories of consciousness, offering complementary insights. According to Global Workspace Theory (GWT) (Baars, 1988; Dehaene et al., 2006), conscious access depends on globally broadcasting information across distributed networks. Seizures can disrupt long-range cortico-cortical and cortico-thalamic synchrony (Bartolomei and Naccache, 2011), preventing networks from achieving the differentiation and complexity needed for conscious processing and producing, in some occasions, overlapping, looping, or accelerated temporal phenomenology. Similarly, Integrated Information Theory (IIT) posits that consciousness reflects integrated information (Tononi, 2004; Oizumi et al., 2014), with hierarchical and parallel neural timescales contributing to both differentiation and integration. Seizures transiently may perturb this balance, revealing latent capacities for complex or multi-component temporal awareness.

Empirical evidence supports this framework. Clinical metrics such as the Consciousness Seizure Scale (CSS), the Ictal Consciousness Inventory (ICI), and the Seizure Perception Survey (SPS) link disruptions in hierarchical network synchronization and oscillatory coupling to graded alterations in consciousness (Nani and Cavanna, 2014; Baglivo et al., 2024). Even during profound alteration of consciousness, patients often retain accurate perception of seizure onset and duration, and reviewing video-EEG recordings can be enlightening (Campora and Kochen, 2016). These findings suggest that subjective experience persists during seizures in structured, measurable ways, and that hierarchical and parallel timescales contribute to both differentiation and integration of temporal information (El Youssef et al., 2022; Baglivo et al., 2024).

Finally, cultural and ecological dimensions modulate temporal cognition. Cross-cultural variation demonstrates that temporal perception is contextually and socially influenced (Fulmer et al., 2014; Sircova et al., 2014; Ellen, 2016; Lee et al., 2017; Ssorin-Chaikov, 2025), supporting the view of time as a multidimensional, plastic construct. Epilepsy provides a natural model for examining how biology, experience, and culture intersect to shape temporal awareness.

The framework generates several empirically testable predictions. First, alterations in intrinsic neural timescales during ictal and peri-ictal states should correlate with specific features of subjective temporal reports, including temporal dilation, compression, and fragmentation, as assessed through structured behavioral and clinical interviews. Second, changes in oscillatory synchronization and physiological rhythms, including measures of neural coherence, cross-frequency coupling, and autonomic indices, should be associated with distinct alterations in subjective temporal experience. Third, clinical consciousness scales (such as the CSS, ICI, and SPS) should correlate with network-level electrophysiological markers of hierarchical organization, including measures of functional connectivity, signal complexity, and large-scale integration.

Although the present framework is intentionally conceptual, it is not intended to remain purely speculative. Its value resides in generating specific, falsifiable predictions concerning the relationships between subjective temporal experience, hierarchical neural timescales, oscillatory coordination, and network organization during epileptic seizures. In this sense, the proposed framework should be viewed as hypothesis-generating rather than hypothesis-confirming.

Future studies combining intracranial EEG recordings, behavioral time perception paradigms, and validated consciousness scales may directly evaluate these relationships. Such multimodal approaches would allow the systematic mapping between neural dynamics, physiological rhythms, and phenomenological reports across different seizure states.

Overall, temporal consciousness may be understood as a multifaceted construct integrating neural dynamics, phenomenological structure, and sociocultural context. Seizures associated with temporal distortions provide a unique window into the heterogeneity of human temporal experience, suggesting that subjective time emerges from the coordination of multiple interacting neural and bodily processes rather than from a single unitary timing mechanism. Rather than offering a definitive explanatory model, this Perspective is intended to provide an interdisciplinary conceptual scaffold capable of guiding future empirical investigations into the neural mechanisms of temporal consciousness in epilepsy.

## Data Availability

The datasets presented in this article are not readily available because the manuscript is a purely conceptual and theoretical work. No new datasets were generated or collected by the authors. The conclusions are based on the synthesis, interpretation, and integration of previously published peer-reviewed literature spanning neuroscience, cognitive science, neurophysiology, and phenomenological philosophy of time. All supporting evidence derives from existing studies cited in the manuscript, and no original empirical or patient-level data were analyzed. Requests to access the datasets should be directed to gdigennaro@neuromed.it.
